# A new frontier for fat: dietary palmitic acid induces innate immune memory

**DOI:** 10.1097/IN9.0000000000000021

**Published:** 2023-05-15

**Authors:** Amy L. Seufert, Brooke A. Napier

**Affiliations:** 1 Department of Biology and Center for Life in Extreme Environments, Portland State University, Portland, OR, USA

**Keywords:** saturated fatty acid, monocytes, macrophages, palmitic acid, innate immune memory, western diet, ketogenic diet, trained immunity, priming, metabolism, epigenetics, inflammation, chylomicron, ceramide, toll-like receptor, CD36, hematopoietic stem cell, oleic acid

## Abstract

Dietary saturated fats have recently been appreciated for their ability to modify innate immune cell function, including monocytes, macrophages, and neutrophils. Many dietary saturated fatty acids (SFAs) embark on a unique pathway through the lymphatics following digestion, and this makes them intriguing candidates for inflammatory regulation during homeostasis and disease. Specifically, palmitic acid (PA) and diets enriched in PA have recently been implicated in driving innate immune memory in mice. PA has been shown to induce long-lasting hyper-inflammatory capacity against secondary microbial stimuli in vitro and in vivo, and PA-enriched diets alter the developmental trajectory of stem cell progenitors in the bone marrow. Perhaps the most relevant finding is the ability of exogenous PA to enhance clearance of fungal and bacterial burdens in mice; however, the same PA treatment enhances endotoxemia severity and mortality. Westernized countries are becoming increasingly dependent on SFA-enriched diets, and a deeper understanding of SFA regulation of innate immune memory is imperative in this pandemic era.

## 1. Introduction

The innate immune system acts as an efficient non-species-specific shield against infection that thwarts a diverse array of pathogens, and has recently been shown to be highly influenced by the nutritional milieu of the host. Specifically, in humans, the innate immune response has been adapting to constantly changing dietary patterns and, more recently, a variety of diets with enriched saturated fatty acids (SFAs). Decades of research have been dedicated to understanding how SFAs modulate innate immune cell function and inflammatory capacity ^[1,2]^; however, it has only recently been described that enriched dietary SFAs can induce innate immune memory.

Innate immune memory is characterized by epigenetic and metabolic changes within macrophages and monocytes induced by a primary inflammatory stimulus that leads to an enhanced (trained immunity or priming) or decreased (tolerance) response to a secondary inflammatory stimulus ^[3]^. Trained immunity and priming are primitive adaptations of innate host defense that results from exposure to a primary inflammatory stimulus, and leads to a faster and greater inflammatory response to a secondary homologous or heterologous challenge ^[4]^. Hallmark features of trained immunity specifically include metabolic alterations that induce long-lasting epigenetic changes within innate immune cells, remodeling of the hematopoietic stem cell (HSC) compartment that allows for a sustained augmented response from developing myeloid cells, an amplified inflammatory response to pathogenic infection, and transmission of epigenetic memory across generations ^[5–9]^. In contrast, the effects of priming do not involve long-term epigenetic modifications or HSC remodeling; however, priming does allow a cell to respond more strongly to secondary inflammatory stimuli, but only if the secondary stimulus takes place before the cell returns to basal inflammation and homeostasis ^[3]^. The most studied inducers of trained immunity are the Bacillus Calmette-Guerin (BCG) vaccine and the fungal antigen, β-glucan. BCG and β-glucan induce cross-protection against heterologous pathogens in humans and in mice, respectively, including cross-protection against severe acute respiratory syndrome coronavirus 2 (SARS-CoV-2) ^[4,10]^. Trained immunity is also known for its dual nature, and can be beneficial or detrimental to the host depending on the disease context and inflammatory status.

There is little known about the impact of dietary SFAs on innate immune memory; however, SFA-enriched diets have been shown to induce long-lasting impacts on innate immune inflammation and microbial infection in mice, via remodeling of the HSC compartment in the bone marrow ^[11,12]^. Specifically, enriched dietary palmitic acid ([PA]; C16:0), the most prevalent SFA found in the diet and circulating in human blood, has been shown to induce trained immunity within myeloid cells, and plays an important role in both homeostasis and infection in vivo ^[1,12,13]^. PA is known to enhance toll-like receptor (TLR)-dependent inflammation by inducing ceramide metabolism, and sensitizes innate immune cells to subsequent TLR stimulation ^[12,14–16]^. During endotoxemia (lipopolysaccharide [LPS]-induced acute systemic shock), mice pre-exposed to PA exhibit enhanced circulating inflammation, disease severity, and mortality compared to vehicle (Veh)-treated mice and showed significantly greater clearance of fungal and bacterial infections compared to Veh-treated mice ^[12,13]^. In an obese mouse model of weight cycling, the adipose tissue (AT) environment skewed resident macrophages toward heightened inflammatory response to microbial stimulation, and this was comparable to a bone marrow-derived macrophage (BMDM) model that used PA pre-treatments to enhance LPS stimulation ^[17]^. The molecular details of SFA-induced and/or PA-induced trained immunity in vitro and in vivo have not been fully defined; however, in light of these exciting results, we will review here the specific relationship between PA and innate immune memory, and discuss the implications of our known data and the need for future investigations that will elucidate the intricate dynamics of this relationship.

## 2. PA mediates macrophage metabolism and function

Trained immunity in macrophages is accompanied by direct crosstalk between metabolites from the Krebs cycle and histone modifying enzymes necessary for enhanced transcription of pro-inflammatory cytokines via epigenetic mechanisms ^[18]^. It is well known that specific metabolic pathways mediate the transcription and release of pro- and anti-inflammatory cytokines by macrophages ^[19,20]^. Glycolytic metabolism in the cytosol is upregulated in pro-inflammatory macrophage responses, and is accompanied by breaks in the Krebs Cycle that allow for accumulation of metabolites that also support the Warburg effect (ie, aerobic glycolysis) ^[21–23]^. In contrast, oxidative phosphorylation in the mitochondria is upregulated, and the Krebs cycle remains intact, in order to support anti-inflammatory macrophage responses ^[19]^. The plasticity of macrophage inflammatory polarization is governed by tightly regulated metabolic pathways that can be disrupted by excessive exposure to dietary SFAs, including PA ^[12,16,24]^.

PA is the most common SFA found in the human body (20%–30% total FAs) and is enriched in meat, dairy products (50%–60% of total fats), and is nearly 30% of total fats in breast milk ^[2,25]^. The western diet (WD) contains high levels of FAs, specifically PA, and sucrose; the ketogenic diet (KD) is exclusively enriched in FAs, and depending on the dietary structure, it may contain excess PA content. In addition to dietary sources, PA can be synthesized endogenously throughout the mammalian body from other FAs, carbohydrates, and amino acids ^[2]^. PA is known as an immunomodulatory molecule, and it has the capacity to regulate inflammatory processes of innate immune cells, including monocytes, macrophages, and neutrophils ^[12,16,26]^.

Historically, it was believed that PA was a ligand for TLR4 and induced TLR4-dependent inflammatory cytokine production ^[27]^. However, an elegant study has recently shown that PA is not a ligand for TLR4, but enhances activation of multiple TLR signaling pathways and subsequent NF-κB-dependent transcription of inflammatory cytokines through c-Jun *N*-terminal kinase (JNK) activation that is dependent on mitochondrial metabolic regulation via mammalian target of rapamycin (mTOR) ^[24]^. These studies were conclusive in determining PA is not a TLR4 ligand, but much still remains to understand how PA is metabolically enhancing TLR-dependent inflammation in macrophages.

Importantly, when there is an excess of PA in the diet, it is reflected in an increase of free PA systemically ^[1,28]^. When macrophages and monocytes are exposed to excess free PA, it is taken up through the membrane scavenger receptor, CD36. CD36-dependent intake of PA can lead to lipid accumulation and modulation of signaling through metabolic dysfunction, including suppressing AMPK activation ^[29,30]^. It has been found that genetic loss of *Cd36* renders murine macrophages insensitive to some TLR2 ligands and induces hyper-susceptibility of mice to *Staphylococcus aureus* infection ^[31]^. Considering this, inhibition of free PA uptake by genetic deletion of *Cd36* leading to a dampened inflammatory response may contribute to enhanced susceptibility to infection and decreased TLR2-mediated inflammation. Currently, it is unknown if CD36 plays a role in PA-induced innate immune reprogramming, but it is clearly required for PA-dependent inflammatory phenotypes.

After CD36-dependent uptake of PA by the macrophage, PA is converted into phospholipids, diacylglycerol (DAG), and ceramides reviewed here ^[1]^; however, in the presence of excess PA, TAG synthesis is stalled at the DAG stage causing accumulation in the cell ^[1]^. Both DAG and ceramides have been shown to enhance TLR-mediated signaling cascades in macrophages and subsequent activation of NF-κB ^[1]^. Specifically, in the presence of excess PA, macrophages enhance expression of adipose fatty acid binding protein, which acts as a PA chaperone during uptake to enhance ceramide synthesis ^[32]^.

Ceramide is a bioactive sphingolipid (SGL) with cell signaling capabilities, and there are three metabolic pathways that can lead to intracellular ceramide synthesis: the de novo pathway, sphingomyelin (SM) hydrolysis, and the endosomal salvage pathway ^[33]^. Stimulating macrophages with LPS induces ceramide synthesis via SM hydrolysis ^[34]^. In contrast, de novo ceramide synthesis in the presence of excess PA is important for increasing TLR4 activation and cytokine production in primary peritoneal macrophages; however, ceramide produced via SM hydrolysis was shown to regulate LPS-induced interleukin 6 (IL-6) secretion in PA-treated RAW macrophages ^[14,15]^. In addition, our laboratory recently showed, using primary BMDMs, that de novo ceramide synthesis is required for PA-induced hyperinflammation in response to LPS; moreover, inhibiting de novo ceramide synthesis completely abolished the hyper-inflammatory impact with respect to tumor necrosis factor (TNF), however only partially inhibited IL-6-mediated and IL-1β-mediated hyperinflammation ^[12]^. Future studies will benefit from determining precisely which pathway(s) of ceramide synthesis may be responsible for driving innate immune cell reprogramming and which proteins and signaling pathways are targeted by ceramide, as any of these have the potential to be therapeutic targets for inflammatory diseases driven by ceramide.

Together, these data conclude PA is not a TLR ligand, but enters macrophages through CD36, and subsequently mediates metabolism to enhance TLR-dependent cytokine production, which may play a critical role in regulating host response to infection and inflammatory diseases. Importantly, the term excess dietary PA is subjective, and likely depends on disease context, and the ability of the host to regulate homeostasis between endogenous and exogenous PA levels; however, physiologically relevant serum PA concentrations have been mimicked in vitro and in vivo to show a significant impact on metabolic pathways that alter macrophage inflammation ^[12,14,15,24,35]^. To target PA metabolism of macrophages therapeutically, future studies should determine the specific threshold of intracellular PA levels that, when exceeded, can lead to metabolic and inflammatory dysregulation.

## 3. Exogenous PA and trained immunity

Trained immunity is a feature of innate immune cells that illuminates their capacity for memory via non-specific stimulation ^[4]^. The training phenomenon is unlike memory exhibited by adaptive immune cells, in that it requires specific metabolic rewiring to induce epigenetic modifications that can last for weeks to years ^[4]^. Trained immunity has been studied mostly in the context of vaccination with BCG, or microbial ligand stimulation using the fungal molecule β-glucan, but recently dietary products have been shown to elicit memory capability within innate immune cells ^[6,8,11,12]^. It has recently been proposed that exogenous PA is capable of altering the metabolic and epigenetic landscape of monocytes and macrophages in order to initiate a non-specific, hyper-inflammatory memory response to secondary stimulation with a microbial ligand ^[12]^. Although the precise impact of PA-mediated metabolism on the monocyte/macrophage epigenome has yet to be described, numerous features of innate immune memory, specifically trained immunity, have been revealed by studies involving exogenous PA treatments in vitro and in vivo.

Many studies show the inflammatory impact of combined PA and TLR ligand treatment on monocytes and macrophages; however, few studies show the effect of pre-treating cells with PA followed by TLR agonist or microbial challenge (Table 1). Pre-treatment experiments allow to understand if the PA-dependent metabolic and epigenetic changes can alter short-term or long-term inflammatory response to secondary stimulation with a microbial challenge (trained immunity). Pre-treatment can be a model for a host that is exposed to excess dietary PA and then challenged by an infection, and these studies can inform how PA may be involved in the induction of innate immune memory.

**Table 1.** The impact of PA on innate immune inflammatory responses and memory.

Conditions	Responses	Biological effects	Models used	References
Simultaneous	Priming	Enhanced ceramide	Primary and immortalized mouse macrophages	^[14,15,36]^
PA + LPS (in vitro)		Enhanced TNF/IL-6 secretion		
		JNK dependent		
PA pre-treatment + LPS (in vitro)	Trained immunity	Enhanced TNF/IL-6/IL-1β secretion, all ceramide dependent	Primary mouse macrophages; immortalized human and mouse macrophages	^[12,17,35,37]^
		Role of mitochondria and MAPK signaling		
PA pre-treatment + LPS (in vivo)	Trained immunity	Enhanced circulating inflammation; decreased survival	Wild-type female BALB/c mice	^[12]^
PA pre-treatment + infection (in vivo)	Trained immunity	Enhanced microbial clearance	*Rag*^−/−^ mice + *Candida albicans* infection; wild-type female BALB/c mice + *Brucella abortus* infection	^[12,13]^

IL-6, interleukin 6; JNK, c-Jun *N*-terminal kinase; LPS, lipopolysaccharide; MAPK, mitogen-activated protein kinase; PA, palmitic acid; TNF, tumor necrosis factor.

It was recently shown that immortalized human and mouse macrophage cell lines that were pre-treated with exogenous PA exhibited enhanced LPS-induced TNF secretion ^[37]^. Schwartz et al showed, in THP-1 monocytes, that the hyper-inflammatory response to LPS following PA-pre-treatment was dependent on ceramide-mediated activation of protein kinase C and mitogen-activated protein kinase signaling pathways ^[35]^. While the mechanism for augmented TNF secretion was not defined by Fang et al, the hyper-inflammatory effect of PA-pre-treatment was associated with enhanced phosphorylation of p38 and JNK, and enhanced expression of carnitine palmitoyltransferase1A, an enzymatic shuttle on the outer membrane of mitochondria that facilitates uptake of activated fatty acids into the mitochondrial matrix for β-oxidation ^[37]^. These data suggest that mitochondrial metabolism plays an important role in the hyper-inflammatory impact of PA-pre-treatment and subsequent challenge with LPS in macrophages ^[35,37]^. These studies, however, did not distinguish if PA pre-treatment was inducing priming or trained immunity, but they did suggest that PA can alter macrophages to respond more acutely to secondary LPS challenge.

Our team recently showed that PA-pre-treatment of BMDMs subsequently challenged with LPS enhanced TNF, IL-6, and IL-1β release; this was dependent on ceramide synthesis, and reversible when BMDMs were pre-treated simultaneously with both PA and the monounsaturated FA (MUFA) that diverts ceramide synthesis in the presence of PA, oleic acid (OA) ^[12]^. Additionally, the synergistic effect of PA and LPS was shown in primary mouse peritoneal macrophages; however, only simultaneous treatment was used to show significantly enhanced de novo ceramide synthesis, and significantly enhanced TNF and IL-6 secretion ^[14]^. Thus, they could not conclude if this was trained immunity. This effect of combined PA and LPS treatment was recapitulated in RAW murine macrophages to show that ceramide mediates LPS-induced IL-6 secretion via JNK phosphorylation; interestingly, this process was regulated by fatty acid transporter 1, and the requirement of CD36 was not shown ^[36]^.

Although these studies have been immensely important in describing the effect of PA on inflammation, it is still unclear if PA is inducing priming or trained immunity induced in macrophages. Thus, future studies regarding the role of PA in innate immune memory should determine the following: (1) The time point of initial inflammatory release in primary stimulation of macrophages with PA; (2) a return to basal inflammation; and (3) hyperinflammation upon secondary stimulation. Together, these outcomes would bolster the hypothesis that PA induces macrophage-trained immunity in vitro.

In vivo, exogenous-free PA has been shown to play an important role during infection. Specifically, in *Rag1*^−/−^ mice that lack adaptive immunity, an intraperitoneal (i.p.) injection of a PA solution 12 hours prior to intravenous (i.v.) infection with *Candida albicans* lead to a significant decrease in kidney fungal burden, compared to infected mice only pretreated with a Veh solution ^[12]^. This suggests that PA-induced memory mediates microbial clearance, and this is dependent on innate immune cells. Further, mice injected i.p. with a PA solution 12 hours prior to LPS-induced endotoxemia show significantly enhanced *Tnf* and *Il-6* expression in the blood within 5 hours post-LPS ^[12]^. Importantly, blood draws were taken immediately before LPS injections to show that baseline inflammation was not upregulated in PA-treated mice, suggesting that LPS-induced cytokine expression in the blood was not a priming effect induced by PA, but rather a trained immunity phenomenon ^[12]^.

Further, in a study by Reyes et al, defining the role of exogenous PA in *Brucella abortus* infection in mice, oral gavage of PA was shown to significantly reduce splenic bacterial burden when administered for 3 days before, and during 14 days of *B. abortus* infection following an i.p. challenge ^[13]^. This was accompanied by a suppression of serum IL-10; however, the mechanism underlying enhanced bacterial clearance induced by PA remains unknown. It is not entirely clear whether these data represent PA-induced priming or trained immunity, because there was no resting period after 3 day PA exposure, or determination of basal inflammatory status before infection with *B. abortus*. The results are still compelling, and to our knowledge, they are the first to show the impact of dietary PA on the clearance of pathogenic bacteria in vivo. Lastly, as mentioned previously, canonical-trained immunity induced by β-glucans or BCG vaccine leads to long-term metabolic and functional reprogramming of myeloid cells, and relies on epigenetic alterations for sustained inflammatory capacity. Remarkably, PA-induced memory was shown to elicit long-lasting immune reprogramming in vivo using an LPS-induced endotoxemia mouse model ^[12]^. Specifically, 9 daily i.p. injections of PA followed by a 7-day resting period and subsequent LPS challenge led to enhanced endotoxemia severity and mortality compared with mice injected with a Veh solution ^[12]^. PA-injected mice showed significantly increased hypothermia compared with Veh-injected mice, and while mortality was enhanced in PA-injected mice, this survival defect was not significant compared with mice injected with PA. Thus, PA exacerbates endotoxemia severity, but this is not sufficient to significantly decrease survival in an endotoxemia mouse model ^[12]^. These data suggest that PA regulates epigenetic modifications that persist beyond the time points of PA exposure, and these alterations adversely impact the ability of the host to regulate body temperature in response to LPS challenge. Thus, PA induces trained immunity in this context, and not priming. Additional experiments will be required to determine whether PA depends solely on innate immune memory mechanisms to exert the long-term adverse effects described here.

Together, these studies build a compelling case that exogenous PA modulates microbial-induced inflammation and clearance in vitro and in vivo. Importantly, the effects of PA exposure in vivo are long-lasting, and PA-exposed mice do not exhibit heightened circulating inflammation before infection, indicating that PA is inducing trained immunity and not priming. The intracellular mechanisms of PA-induced memory and the subsequent augmenting effects on microbial ligand stimulation are not fully characterized. Although intracellular ceramide has been shown to mediate PA-induced memory in macrophages, it is still unclear how the metabolism of PA may lead to epigenetic alterations that ultimately modify the expression of inflammatory response genes.

## 4. Enriched dietary PA, trained immunity, and obesity

Thus far we have described experimental data involving exogenous PA and its effects on monocyte and macrophage metabolism, inflammation, and memory, in addition to in vivo models of PA injections (i.p.) and oral gavage. Next, we outline the impacts of enriched dietary PA consumption on the development of innate immune cells in the bone marrow, and how this may contribute to long-term innate immune memory, specifically trained immunity.

Recent studies from our group and others have begun to understand the effect of excess chronic dietary PA on systemic response to microbial challenge and macrophage function ex vivo. Christ et al found that atherosclerotic mice (*Ldlr*^−/−^ C57BL/6) fed WD for 4 weeks resulted in a hyper-inflammatory response when challenged with LPS ex vivo ^[11]^, suggesting exposure to excess dietary PA may be a factor in influencing the inflammatory capacity of myeloid cell populations in vivo. More recently, we have reported wild-type (WT) mice fed WD and KD, both enriched in PA, exhibit increased systemic inflammation in response to endotoxemia, a single-intraperitoneal injection of LPS ^[12,38]^. Importantly, we show that this enhanced systemic inflammation in response to LPS is independent of glycolytic shock and the diet-induced microbiome, further suggesting that enriched dietary PA is leading to an enhanced response to TLR4 agonist LPS ^[12]^. Together, these studies suggest a direct link between enriched dietary PA and trained immunity in vivo.

A hallmark of canonical-trained immunity in vivo is the skewing of the HSC compartment toward increased myeloid cell production and enhanced inflammatory capacity ^[8,11]^. It has been shown that 4 weeks of WD administration was sufficient to alter HSC populations within the bone marrow of atherosclerotic mice; and after reverting back to a standard chow for an additional 4 weeks, stem cell progenitors remained skewed toward developing monocytes with hyper-inflammatory potential ^[11]^. More recently, we published that WT BALB/c mice on WD for 2 weeks do not exhibit altered HSC populations ^[12]^. Importantly, these studies may disagree due to the use of different mouse models (*Ldlr*^−/−^ C57BL/6 vs WT BALB/c) and the length of diet administration; more follow-up studies are required to understand this bifurcation. However, we have additionally shown that exposure to a PA-enriched KD skews the HSC compartment to develop significantly enhanced populations of long-term and short-term HSCs and multipotent progenitors ^[12]^. Importantly, no one has shown whether PA is responsible for the HSC skewing in either of these models.

As mentioned previously, trained immunity can last days to years after initial induction with a primary inflammatory stimulus. While the endurance of dietary PA-induced memory remains unknown, the impacts exhibit hallmark features of trained immunity, including (1) the induction of long-term hyper-inflammatory capacity toward microbial stimuli, (2) the low basal inflammatory status shown in vitro and in vivo following PA treatments before secondary stimulation, (3) the enhanced clearance of microbial infection in vivo, and (4) the induction of HSC remodeling that mimics HSC remodeling in BCG-induced trained immunity ^[8]^.

Alongside a growing body of evidence that shows dietary SFAs disrupt metabolic and inflammatory homeostasis, many disorders characterized by immunometabolic dysfunction are associated with excessive WD exposure, including obesity, type II diabetes, cardiovascular disease, neurodegenerative, and autoimmune diseases—all filed under the umbrella term, metabolic syndrome ^[39]^. While obesity is not solely a diet-driven disease, weight gain is the most observable side effect, and as a result, weight gain is often misrepresented as the driver of enhanced susceptibility to poor outcome during infectious disease. However, our laboratory has shown that WD-induced weight gain does not correlate with endotoxemia severity and mortality in WT mice ^[38]^. This suggests that dietary constituents of the WD, and not the ensuing weight gain, are the drivers of innate immune reprogramming and enhanced susceptibility to secondary microbial exposure. A paradox currently exists in the literature that shows a diversity of severity and mortality outcomes in obese sepsis patients, whereby obesity appears to be protective in some studies, whereas others show that obesity correlates with exacerbated sepsis outcome ^[40]^. Sepsis is characterized by widespread inflammatory dysregulation, brought on by an infection that has entered the bloodstream, and it can be induced by a variety of microbial pathogens ^[41]^. Interestingly, obese populations have been shown to exhibit variability in serum PA levels and also metabolic health—indicated by the presence or absence of insulin resistance ^[42,43]^. For example, a subset of the obese population is considered metabolically healthy if they do not exhibit insulin resistance, and this population exhibits significantly lower levels of PA in their serum compared with the serum of metabolically unhealthy obese individuals ^[42]^. We believe that this correlation between PA levels and metabolic health in obesity, in addition to the obesity paradox in inflammatory disease outcome in humans, align with the variability in disease outcome that PA-induced memory exhibits in mouse studies of trained immunity. Specifically, the widely acknowledged duality of trained immunity is a key feature of this memory phenomenon that explains the ability of SFAs like PA to impose beneficial, or detrimental, inflammatory disease outcomes that are likely dependent on the type of microbial species acting as the secondary stimuli and disease trigger.

In harmony with numerous obesity studies that highlight the metabolic and inflammatory dysregulation that occurs alongside diet-induced obesity, weight cycling was recently considered in Caslin et al’s work using a unique BMDM model, whereby AT harvested from weight-cycled mice (high fat-diet-induced obesity followed by low fat-diet-induced weight loss within a 9-week period) was used to condition culture media for BMDMs. They showed that, similar to BMDMs given a 24 hours PA pre-treatment, the BMDMs given AT-conditioned media from weight-cycled mice were hyperresponsive to subsequent LPS treatments (TNF and IL-6). They also showed that AT macrophages (ATMs) from weight-cycled mice exhibited enhanced TNF/IL-6 secretion with ex vivo LPS stimulation, compared with ATMs from lean mice ^[17]^. This is important because it suggests that secretions that are put out into the AT environment, not only during obesity, but also—and surprisingly even more so—following subsequent weight loss are capable of reprogramming the immune response of primary macrophages. Moreover, the AT environment likely includes PA as a result of adipocyte lipolysis; however, whether or not PA was the driver of enhanced inflammatory responses shown in their experiments is unknown. How this may impact the host response to subsequent infection is currently of paramount importance, as metabolic syndrome and increased risk of pathogenic exposure are both increasingly evident public health concerns.

Although it was not the main finding of Caslin et al’s weight cycling study, it is notable that their use of a canonical-trained immunity model using BMDMs showed that PA pre-treatment, followed by a 6-day resting period, not only enhanced TNF/IL-6 secretion in response to subsequent TLR ligand stimulation but also to β-glucan stimulation ^[17]^. Importantly, this hyper-inflammatory response to β-glucan was not present in BMDMs lacking TLR4, suggesting that although PA does not signal through TLR4, PA-induced memory still requires TLR4 in order to enhance the innate immune response to fungal molecules specifically. To our knowledge, this is the first study that shows PA-induced memory requires TLR4 in order to augment the innate immune response to β-glucan. Moreover, our laboratory recently showed that *Rag1/2^−/−^* mice given i.p. PA injections 12 hours before infection with *Candida albicans*, a fungal pathogen that is recognized by innate immune cells via β-glucan, exhibited enhanced clearance of kidney fungal burden compared with mice only pre-exposed to a Veh solution ^[12]^. Although the studies surrounding PA-induced immunomodulation are still in their infancy, the ability of PA to induce long-lasting, hyper-inflammatory responses to microbial ligands isolated from bacterial and fungal pathogens via innate immune pattern recognition receptors may lend insight into the diversity of diet-induced disease responses currently referred to as paradoxical in the literature. For example, the opposing disease outcomes shown in mice exposed to TLR ligands such as LPS, or Dectin-1 ligands like β-glucan, following the induction of PA-induced innate immune memory, may indicate that not only does the amount and duration of PA exposure play a role in disease outcome following secondary exposure to microbial ligands, but also the specific microbial species themselves that act as the secondary inflammatory trigger may determine whether or not PA-induced memory will lead to a beneficial or detrimental disease outcome. Future studies should identify the infectious disease contexts that may benefit from prior innate immune modulation via dietary PA, in addition to the infectious agents that may exacerbate the host inflammatory response following dietary PA exposure. Moreover, personalized dietary intervention that uses serum PA measurements may improve inflammatory disease treatments, and prevention strategies for individuals susceptible to infectious disease.

A common co-morbidity in obese patients is the leaky gut that often occurs following a chronic low-fiber diet. Commensal gut microbes metabolize dietary fiber, and during fiber deficiency they will instead feed on the mucus lining the gut, leading to a breach in the epithelial barrier, followed by a release of microbiota-associated LPS into the bloodstream, and potentially life-threatening opportunistic infections ^[44,45]^. Microbial molecules like LPS that breach this barrier will first induce a pro-inflammatory effect known as metabolic inflammation, or metaflammation, characterized by a chronic state of heightened pro-inflammatory cytokine levels in the blood and tissues ^[46]^. Chronic metaflammation can tolerize innate immune cells to microbial ligand exposure, and render them ineffective in response to infection, which can promote immunoparalysis following acute systemic infection during sepsis ^[38]^. Thus, another paradox may arise in considering the induction of trained immunity via dietary PA, whereby exposure to PA may initially cause hyperinflammation in response to secondary ligand exposure; however, if PA exposure if excessive and chronic, innate immune tolerance may play a role in dampening the host response to infection. We believe that serum lipidomics and personalized nutrition will be necessary in order to balance levels of PA and PA-saturated lipids in the blood of individual patients, to prevent an imbalance in that may skew patients toward hyper-responsive or hypo-responsive inflammatory response during specific disease contexts (local infection vs systemic infection or sepsis).

## 5. PA from the mouth to the bone marrow

**Figure 1.**
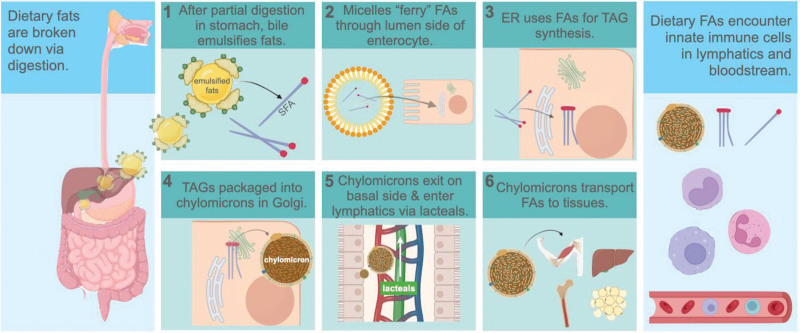
**Pathway of dietary fatty acids following digestion.** Following enzymatic digestion in stomach, (1) bile salts from the gall bladder emulsify FAs and MAGs during entry into the duodenum. (2) FAs greater than 14-carbons in length are packaged into micelles that ferry them into enterocytes from the lumen. (3) FAs are resynthesized into TAGs in the ER, followed by (4) repackaging into chylomicrons within the Golgi. (5) Chylomicrons are carried in vesicles through the lymphatics within chylomicrons, and may encounter monocytes, macrophages, and neutrophils. Figure was created with biorender.com (https://www.biorender.com/). ER, endoplasmic reticulum; FA, fatty acid; MAG, monoacylglyceride; TAG, triacylglyceride.

To determine the holistic impact of dietary PA on innate immune memory, we must also consider the pathway that PA follows after digestion, and the byproducts of PA metabolism that may impact innate immune cells enroute to various tissues for energy or storage purposes. PA and its metabolic byproducts encounter innate immune cells before being distributed to tissues, because many fats, depending on their size and diffusion capability, circulate in lipoprotein structures throughout the lymphatics even before entering the bloodstream ^[47]^. Dietary PA digestion, absorption, and mobilization to tissues is complex compared to that of carbohydrates and proteins, which are transported directly to the liver via the superior mesenteric and hepatic portal veins immediately following digestion, and absorption from the small intestine into the blood circulation (Figure 1) ^[48,49]^.

The basic structure of PA is a 16-carbon chain saturated with hydrogen, and thus too large to diffuse from the small intestine directly into capillaries leading to the blood ^[50]^. PA requires the emulsification action of bile, and ferrying within bile micelles toward the luminal epithelium of the small intestine ^[51]^ (Figure 1). Diffusion can then occur from the micelles that act as PA carriers, into the enterocytes of the small intestine, where they are broken down, reassembled into triglycerides (TAGs), and packaged into chylomicrons before being taken up by lymphatic lacteals ^[52]^. Chylomicrons are structures with a lipid membrane that contain hydrophobic lipids and proteins internally, and they enter into the lymphatics via lacteals, small ducts at the ends of lymphatic vessels within microvilli of the small intestine. After circulating through the lymph, PA-carrying chylomicrons enter the venous circulation via the thoracic duct and the lymphatic ducts near the subclavian veins. Then, the chylomicrons travel through the heart and lungs and are distributed to the remaining peripheral tissues including AT, skeletal muscle, bone marrow, and finally the liver, where they can be used for energy for various metabolic processes, incorporated into lipid membranes for cells and organelles, or stored as TAGs for later use ^[52]^. The CO_2_ waste from PA digestion is excreted via the respiratory system, and the term chylomicron remnants is often used to describe the final remaining TAGs that are taken to the liver after distribution throughout the rest of the body.

There are many opportunities for innate immune cells to encounter PA, which can be in the form of free fatty acids (FAs), TAGs, phosphatidylcholines, and SGLs while circulating in the lymphatics and blood to be distributed to brain, skeletal muscle, adipose, bone marrow, liver, and spleen tissues for energy use or storage ^[53]^. Of particular importance is in the bone marrow, where HSCs may be reprogrammed by dietary PA, and then later differentiate into more specific progenitor cells with enhanced inflammatory capacity ^[11,12,54]^. It is important to consider the likelihood that reprogrammed HSCs may then seed tissues during stress or a localized infection, and exacerbate inflammation potentially leading to disruption of inflammatory homeostasis within a tissue or whole organ.

This unique pathway that PA embarks on following digestion, and its ability to be transported within chylomicrons to the bone marrow, is especially important for the long-term nature of innate immune memory. Because of the short lifespan of circulating monocytes (1–3 days) before their differentiation into tissue-specific macrophages, it seems unlikely that these cell types would retain long-term, non-specific memory; however, PA-induced HSC reprogramming may have the potential to harbor epigenetically modified progenitor cells within the bone marrow that can be subsequently recruited to infected tissues, and respond with hyper-inflammatory output to quickly clear infection. Moreover, in the context of dysregulated inflammation, such as in a septic response, trained HSCs would likely provide too much inflammation and exacerbate disease via tissue damage. Considering that dietary SFAs can be transported to the bone marrow and alter HSC populations, the role of dietary PA in bone marrow remodeling is crucial to consider when determining therapeutic intervention strategies for infection and inflammatory diseases among populations that consume PA-enriched diets.

## 6. Plasticity of PA-dependent innate immune memory

There is evidence that PA-dependent innate immune memory induces metabolic and inflammatory changes that are reversible, and in contrast to PA exerting pro-inflammatory effects, certain unsaturated FAs (UFAs) are known to promote an anti-inflammatory outcome. It is now appreciated that PA accumulation can lead to pathophysiological changes and inflammation when there is an imbalance of dietary PA/MUFAs. OA is the second most prevalent FA in the blood next to PA, and it has been shown to induce low levels of inflammation in macrophages in vitro at certain concentrations ^[55]^. However, OA is considered to be an anti-inflammatory MUFA, due to its ability to counteract or reverse the inflammatory impacts of SFAs in vitro ^[55]^. For example, in mouse peritoneal macrophages, OA reverses PA-induced and stearic acid (SA)-induced IL-1β secretion ^[56]^. In vivo, OA injections have been shown to mitigate the endotoxemia severity and mortality that is exacerbated in KD-fed mice ^[12]^. The polyunsaturated FA (PUFA), docosahexaenoic acid, has also been shown to counteract the inflammatory effects of PA in RAW macrophages and BMDMs, displaying a specific target against de novo synthesis of ceramide ^[57–59]^. Thus, specific ratios of SFAs to MUFAs, or SFAs to PUFAs in the blood, bone marrow, and peripheral tissues, such as liver, spleen, adipose, skeletal muscle, and the central nervous system, will be important to consider when studying the regulatory impact of FAs on innate immune inflammation in vivo.

**Figure 2.**
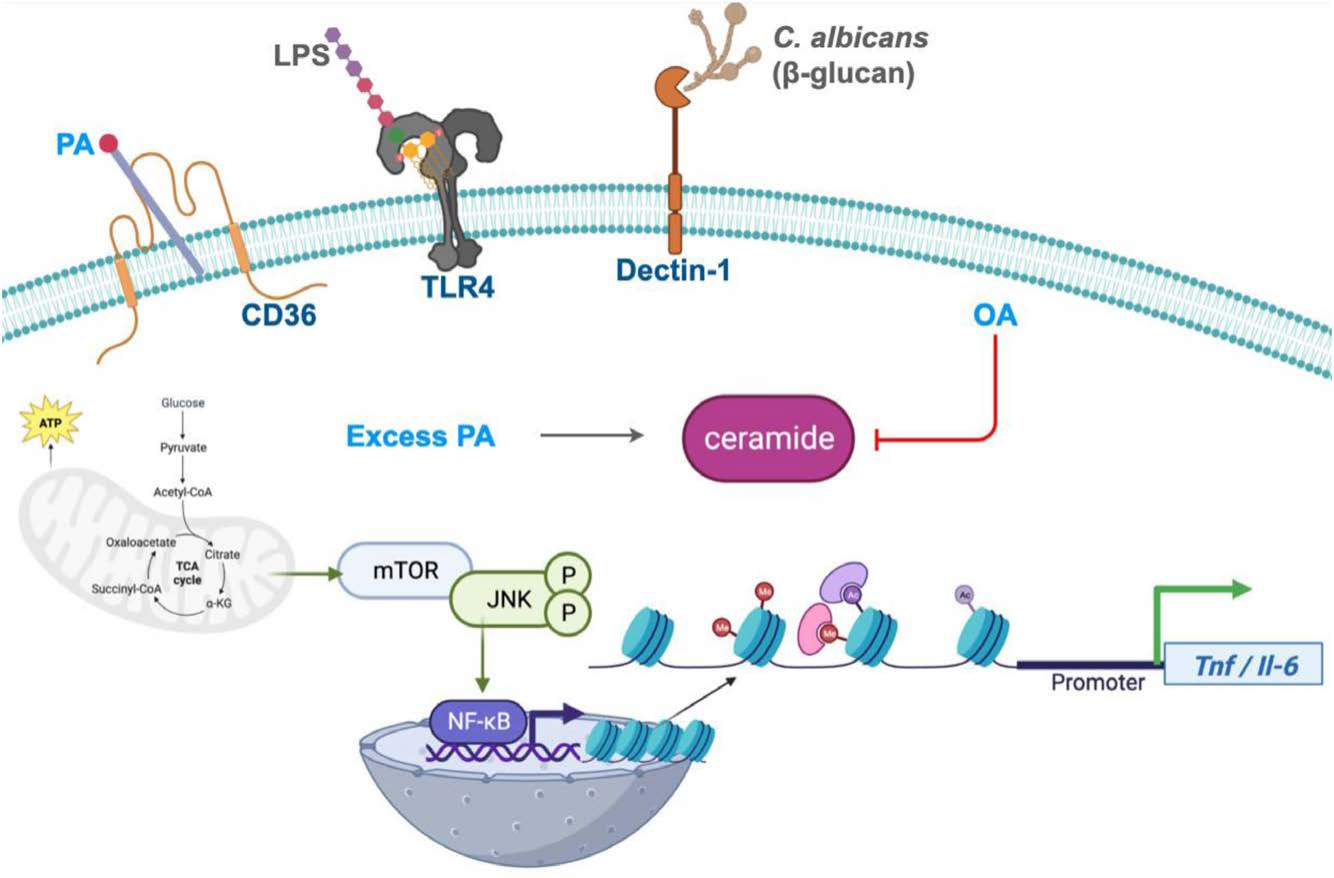
**A dietary rheostat for macrophage inflammatory homeostasis and trained immunity.** PA enters macrophage through the scavenger receptor, CD36; excess PA increases de novo ceramide; subsequent TLR4 stimulation by LPS enhances intracellular ceramide and activates NF-кB; PA metabolic byproducts induce histone modifications at *Tnf/ll-6* promoters; fungal β-glucans signal through Dectin-1 and activate NF-кB; mTOR regulates JNK activation and NF-кB transcription; OA inhibits ceramide and reverses inflammatory polarization. Figure was created with biorender.com (https://www.biorender.com/). JNK, c-Jun *N*-terminal kinase; LPS, lipopolysaccharide; mTOR, mammalian target of rapamycin; ; NF-кB, Nuclear factor kappa B; OA, oleic acid; PA, palmitic acid; TLR4, toll-like receptor 4.

It is tempting to consider the possibility that a metabolic rheostat exists between trained immunity and inflammatory homeostasis and that can be controlled and balanced with specific ratios of dietary SFAs to UFAs; if inflammation is required to clear microbial burden, then SFAs may work to upregulate it, and if tissue homeostasis requires restoration after a hyper-inflammatory event, then UFAs may serve to downregulate inflammation before it becomes damaging (Figure 2). If innate immune cells must adapt to a changing dietary environment, they may be required to increase or decrease their uptake of SFAs and UFAs, and perhaps this function is perturbed during excessive and prolonged exposure to SFAs.

## 7. Conclusion: where to next?

The studies we present here provide compelling evidence that PA is an immunomodulating SFA that reprograms the innate immune cell response to secondary inflammatory stimuli in vitro and in vivo, that is, PA induces innate immune memory. Although some outcomes suggest that PA induces priming, more recent investigations show the long-term impact of PA on innate immune inflammatory regulation. This long-term nature of PA reprogramming, in addition to the ability of PA to alter secondary inflammatory outcomes even after basal inflammation has been reached, strongly supports the hypothesis that PA induces trained immunity.

For future in vivo experiments, more long-term studies need to be done to collectively elucidate the following: (1) The physiologically relevant PA concentrations that induce the initial inflammatory response—are they relevant to consumers of SFA-enriched diets? (2) The initial inflammatory response to dietary PA—is it systemic or localized to specific tissues? (3) The minimum and maximum rest periods from PA exposure required to return to inflammatory homeostasis and still maintain innate immune memory reprogramming—how long does the memory last? (4) The specific metabolites and histone modifications that are required for epigenetic reprogramming of innate immune cells—what is the mechanism behind the augmented inflammatory response to secondary stimulation? Addressing these questions will advance our knowledge of PA-mediated trained immunity and how it regulates innate immune homeostasis, inflammation, and the response to infection.

PA constitutes 20%–25% of human breast milk, and if PA induces trained immunity, it is interesting to postulate that enhanced maternal dietary intake of PA may induce trained immunity in nursing neonates ^[60]^. Enhanced PA within maternal milk may influence the capacity of a neonate to induce inflammation and or enhance protection against microbial challenge. Interestingly, a study from Du et al shows that maternal WD consumption in mice causes the production of milk that contains excessive PA, ceramide accumulation, and inflammation in nursing neonates ^[61]^. Additionally, this ceramide accumulation and inflammation in nursing neonates was TLR4/2 dependent; however, they did not look into how enhanced PA milk effected neonatal (1) protection against microbes, (2) neonate HSC composition, or (3) neonatal macrophage response to LPS challenge.

Although most immunological studies highlight the impact of PA on monocytes and macrophages, neutrophils may also play a role in SFA-induced inflammation and innate immune memory. For example, neutrophil recruitment and inflammation are enhanced by PA-treated BMDMs through a chemotactic response mechanism. Specifically, PA induces pannexin channel formation and nucleotide release, which then attracts neutrophils to the high-SFA region that macrophages were exposed to ^[62]^. Other dietary SFAs are also known to stimulate the release of neutrophil precursors from the bone marrow of mice into the bloodstream ^[63]^. Importantly, the direct impact that PA has on neutrophil metabolism and subsequent inflammation has not been fully studied. Future studies should focus on illustrating the impact that PA-enhanced neutrophil recruitment may have on inflammatory homeostasis, and the host response to microbial triggers.

The discovery that PA induces innate immune memory, and modulates inflammatory and infection outcomes in mice, is just the beginning of a much greater appreciation for the nuances of dietary-regulated immune function. A deeper understanding of the crosstalk between dietary SFAs and innate immune inflammation is imperative for understanding the long-term impact of diets enriched in SFAs on the host response to infection and inflammatory disease. This will contribute to effective prevention and treatment of diseases exacerbated by excessive SFA intake, advanced personalized nutrition, and a greater sense of agency over our immune health while living in societies heavily dependent on dietary SFAs.

## Conflicts of interest

The authors declare no conflicts of interest.

## Funding

This study was supported by National Institute of General Medical Sciences (NIGMS) grant 5R35GM133804-02 to BAN.
